# Development and validation of an explainable machine learning and nomogram model for early detection and risk stratification of polycystic ovary syndrome: a multicenter study

**DOI:** 10.3389/fendo.2025.1719631

**Published:** 2025-12-17

**Authors:** Bihua Yao, Xingyu Yu, Yunyan Zhang, Jiayan Chen, Xiaotong Zhu, Cheng Zhang, Tong Jijun

**Affiliations:** 1 Laboratory Medicine Center, Department of Clinical Laboratory, The First People’s Hospital of Jiashan, Jiashan Hospital Affiliated to Jiaxing University, Jiaxing, Zhejiang, China; 2 School of Laboratory Medicine and Life Sciences, Key Laboratory of Laboratory Medicine, Ministry of Education, Wenzhou Medical University, Wenzhou, Zhejiang, China; 3 The Key Laboratory of Intelligent Unmanned Systems Software Technology and Applications, Zhejiang Sci-Tech University, Hangzhou, Zhejiang, China; 4 Department of Gynecology, The First People’s Hospital of Jiashan, Jiashan Hospital Affiliated to Jiaxing University, Jiaxing, Zhejiang, China; 5 Laboratory Medicine Center, Department of Clinical Laboratory, Jiaxing Hospital of Traditional Chinese Medicine Affiliated to Zhejiang Chinese Medical University, Jiaxing, Zhejiang, China

**Keywords:** early screening, machine learning, nomogram, polycystic ovary syndrome, Shap, XGBoost

## Abstract

**Background:**

Polycystic ovary syndrome (PCOS) is a common endocrine–metabolic condition in reproductive-aged women, linked to infertility and long-term cardiometabolic risk. Early identification remains challenging because current diagnosis relies on hormone testing and imaging. This research sought to develop and evaluate an interpretable machine learning (ML) model and a simplified nomogram for the early detection of PCOS.

**Methods:**

Data from 1,600 women at the First People’s Hospital of Jiashan were used for model training, with 283 external cases from Jiaxing Hospital of Traditional Chinese Medicine for validation. Twenty-three routine laboratory indicators were analyzed. After LASSO feature selection, seven ML algorithms were compared. The best-performing XGBoost model was interpreted using Shapley Additive exPlanations (SHAP). A logistic regression–based nomogram was developed from the key predictors.

**Results:**

The XGBoost model showed excellent discrimination (AUC = 0.919 internal; 0.923 external). SHAP identified DHEAS, AMH, TG, and age as key contributors. The nomogram also performed well (AUC = 0.901 train; 0.887 test).

**Conclusions:**

This interpretable “XGBoost + SHAP” and nomogram framework provides an accurate, transparent, and practical tool for early PCOS screening and individualized management.

## Introduction

Polycystic ovarian syndrome (PCOS), one of the most common endocrine and metabolic disorders in reproductive-aged women (1), is estimated to affect 8%–13% of the global population (2–4). Its clinical and metabolic manifestations are highly heterogeneous, typically defined by hyperandrogenism, ovulatory dysfunction, and polycystic ovarian morphology, often associated with insulin resistance (5) and metabolic syndrome (6, 7), posing long-term reproductive and metabolic health risks for women (8). The Rotterdam 2003 criteria remain the most widely accepted diagnostic standard, requiring at least two of the three features for diagnosis (9, 10). The 2023 international evidence-based guideline further emphasized the integration of clinical, biochemical, and imaging evidence, recognizing anti-Müllerian hormone (AMH) as an alternative marker for defining polycystic ovarian morphology (PCOM), but not as a standalone diagnostic test (11). Owing to the heterogeneity of PCOS, current diagnosis relies heavily on hormonal assays, menstrual history, and imaging evaluations, which are influenced by timing, instrument variability, and operator experience, resulting in missed or delayed diagnoses, particularly in early or atypical cases (12, 13). Therefore, there is an urgent need for objective, stable, and easily accessible serum-based indicators. Recent systematic reviews have identified AMH, androgens, insulin-resistance-related indices, and lipid-metabolism markers (14–16) as potential key biomarkers, providing new directions for the early identification and risk stratification of PCOS.

The rapid advancement of artificial intelligence (AI) and machine learning (ML) has shifted clinical research paradigms from experience-driven to data-driven (17–23), offering innovative strategies for disease prediction and early diagnosis (23). However, most existing ML studies on PCOS have been limited by small sample sizes or single-center data and have rarely achieved a balance between predictive performance and clinical applicability, hindering clinical translation. Meanwhile, although diagnostic and therapeutic approaches continue to evolve, the etiology and management of PCOS remain complex, and current interventions mainly focus on symptomatic control (24). Hence, novel intelligent diagnostic and decision-support tools are needed to facilitate early risk detection and personalized management.

In this study, we integrated multicenter clinical data encompassing routine hormonal and metabolic indicators to propose a dual-model strategy. First, we developed a high-performance machine learning (ML)- based screening model, PCOS-XGBoost. We applied Shapley Additive exPlanations (SHAP) (25) to elucidate key predictors and their threshold effects, thereby enhancing model interpretability and transparency. Second, we constructed a simplified logistic-regression nomogram to translate complex algorithms into a clinically intuitive tool. Through this complementary framework, the study aimed to strike a balance between predictive accuracy and clinical usability, providing a feasible approach for early PCOS screening and individualized management.

## Materials and methods

### Data source

This retrospective study enrolled 1,600 women who first visited the Department of Gynecology at the First People’s Hospital of Jiashan between January 2021 and January 2025 as the training cohort, and 283 women from the Jiaxing Hospital of Traditional Chinese Medicine between January 2024 and January 2025 as the external validation cohort.

All data were de-identified before analysis. The study followed the Declaration of Helsinki and was approved by both institutional ethics committees (JZYLUN2025–034 and JSYIRB2024-103). As this was a retrospective study, the need for informed consent was exempted.

### Participants

Eligible participants were women aged 18–45 years who had not used medications affecting hormone levels (e.g., oral contraceptives) within the past three months, met at least two Rotterdam 2003 criteria—oligo/anovulation, clinical or biochemical hyperandrogenism, and polycystic ovarian morphology—and had complete clinical and laboratory data.

Patients were excluded if they had missing key data, duplicate records, other endocrine disorders (e.g., congenital adrenal hyperplasia, thyroid dysfunction, or Cushing’s syndrome), severe organic diseases (e.g., endometriosis), were pregnant or lactating, or had recently received hormonal therapy.

### Data extraction and processing

To ensure data quality and model reliability, the raw dataset was thoroughly cleaned by removing records with excessive missing values, outliers, or measurements below the detection limit. In total, 23 clinical parameters were retained for model development, including age, AD, DHT, 17α-OHP, E1, LH, P, T, FSH, E2, PRL, DHEAS, AMH, INS-0h, INS-0.5h, INS-1h, INS-2h, INS-3h, TCH, TG, ApoE, Lp(a), and Glu.

To enhance the consistency and reliability of hormone level measurements, all participants had their hormone levels measured during the follicular phase of the menstrual cycle. This ensured standardized conditions for hormone testing. Testosterone and other hormones were measured using mass spectrometry (MS), providing high sensitivity and accuracy. All hormone measurements were performed by accredited laboratories following strict standard operating procedures.

The Python multiple imputation by chained equations (MICE) approach was used to impute missing data. Highly correlated features (Spearman r > 0.6) were excluded, and all variables were standardized using Z-scores. To address class imbalance, random undersampling was applied, which helped reduce bias and prevent overfitting.

### Feature selection and data partitioning

LASSO regression was used for feature selection, with 10-fold cross-validation and the λ1se criterion to determine the final variables included in the model. A dataset was randomly split into a 7:3 ratio, creating a training set (n = 1,120) and an internal validation set (n = 480). The model’s generalizability was evaluated using an independent external validation set (n = 283).

### Statistical analysis and model development

All analyses were performed in R 4.3.3. Normality was assessed with the Kolmogorov–Smirnov test. Non-normal data are presented as median (IQR) using the Mann–Whitney U test; categorical data as n (%) using the Pearson χ² test. Class imbalance was addressed by random undersampling; data were split 7:3 into training and internal validation sets. The λ1se criterion and LASSO with 10-fold cross-validation were used for feature selection. The objective function was:


$$\hat{\beta_{0}},\hat{\beta }\;=\arg\min\left\{\sum_{i=1}^{n}{\left(y_{i}-\beta_{0}-\sum_{j=1}^{p}\beta_{j}x_{ij}\right)}^{2}\right\}\;\;$$

$$Subject\;to\sum_{j=1}^{p}\left|\beta_{j}\right|\leq \;\lambda$$

### Where λ controls regularization and drives some coefficients to zero

Using the variables selected by LASSO, we trained Light Gradient Boosting Machine (LGBM) (26), Random Forest (RF) (27), Support Vector Machine (SVM), Extreme Gradient Boosting (XGBoost), Decision Tree (DT) (28), K-Nearest Neighbors (KNN), and Logistic Regression (LR) models with tenfold cross-validation. The area under the ROC curve (AUC) was used as the primary performance metric. Among these models, XGBoost achieved the best performance and was further evaluated in the internal and external validation cohorts.

### Model evaluation

The model’s performance was comprehensively evaluated using various metrics, including accuracy, precision, recall, F1 score, sensitivity, specificity, positive predictive value (PPV), and negative predictive value (NPV). To assess clinical applicability, Calibration curves, clinical impact curves (CIC), and decision curve analysis (DCA) were constructed to identify the optimal decision threshold. The generalizability of each model was further tested using both internal and external validation cohorts.

### Model interpretability

XGBoost model predictions were explained using the Shapley Additive exPlanations (SHAP) approach for clarity. Feature importance plots illustrated the overall contribution of each variable, while individual case analyses visualized how key features influenced specific predictions, providing insight into the model’s decision-making process.

### Nomogram construction and validation

Among all models, XGBoost showed the best predictive performance and was selected as the primary model. However, due to its “black-box” nature, direct clinical application may be limited. To improve interpretability, a simplified nomogram was developed using logistic regression based on the key features identified by XGBoost, translating complex model outputs into an intuitive clinical tool for individualized risk assessment.

The nomogram’s performance was evaluated using ROC curves, compared with single predictors, and further verified with DCA and calibration curves. Results demonstrated that the nomogram retained the predictive value of core variables while showing strong robustness and clinical utility.

## Results

### Baseline characteristics

**Figure 1:**
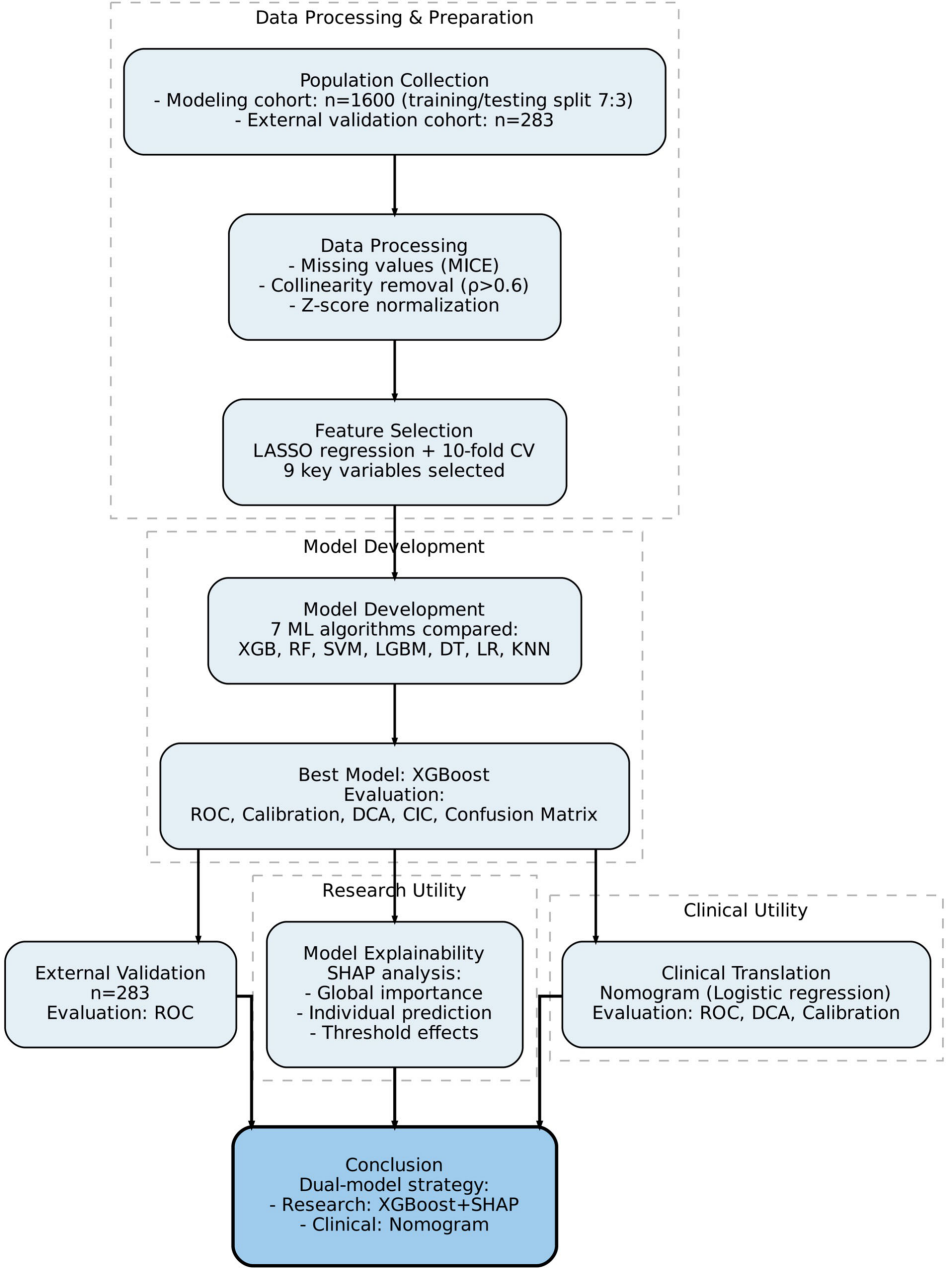
Study the workflow of model development and validation.

A total of 1,600 participants were enrolled, comprising 800 cases of PCOS and 800 non-PCOS cases. The cohort was randomly split into a training set (n = 1,120) and a testing set (n = 480) at a 7:3 ratio. **Table 1** presents the baseline characteristics, while **Figure 1** illustrates the study method.

**Table 1:** Statistics of characteristics of the PCOS and non-PCOS individuals.

Variables	Total (n = 1600) M (Q1, Q3)	Test (n = 480) M (Q1, Q3)	Train (n = 1120) M (Q1, Q3)	p
grouped, n (%)				0.548
0= (non-PCOS)	800 (50%)	234 (48.75%)	566 (50.54%)	
1= (PCOS)	800 (50%)	246 (51.25%)	554 (49.46%)	
age, years	28 (25, 32)	28 (25, 32)	28 (25, 32)	0.399
AD, ng/ml	1.34 (0.96, 1.87)	1.39 (0.96, 1.91)	1.32 (0.97, 1.86)	0.426
DHT, pg/ml	98.06 (49.47, 163.43)	95.04 (47.14, 168.73)	98.2 (50.61, 161.45)	0.700
17α-OHP, ng/ml	0.48 (0.3, 0.73)	0.49 (0.31, 0.72)	0.47 (0.3, 0.74)	0.439
E1, pg/ml	52.06 (34.45, 74.96)	51.12 (35.13, 72.13)	52.45 (34.25, 76.06)	0.587
LH, IU/L	5.3 (3.09, 9.48)	5.19 (3.18, 9.14)	5.41 (3.06, 9.68)	0.583
P, nmol/L	0.33 (0.2, 0.65)	0.31 (0.2, 0.61)	0.34 (0.21, 0.65)	0.162
T, nmol/L	1.05 (0.73, 1.44)	1.1 (0.75, 1.47)	1.03 (0.73, 1.41)	0.186
FSH, IU/L	5.14 (4.12, 6.35)	5.18 (4.08, 6.24)	5.12 (4.13, 6.39)	0.631
E2, pg/mL	45.78 (32.97, 79.84)	44.41 (33.44, 75.64)	46.32 (32.9, 80.65)	0.572
PRL, ng/mL	12.84 (9.21, 18.56)	12.5 (8.69, 17.84)	13.04 (9.47, 18.7)	0.06
DHEAS, μg/dL	189.12 (136.44, 268.74)	191.51 (133.64, 266.98)	187.78 (137.61, 270.73)	0.817
AMH, ng/ml	5.64 (2.87, 9.7)	5.81 (2.98, 9.96)	5.51 (2.78, 9.63)	0.297
INS.0h, mU/L	8.64 (5.8, 12.59)	8.46 (5.64, 12.46)	8.75 (5.87, 12.6)	0.465
INS.0.5h, mU/L	72.48 (47.34, 110.95)	69.91 (45.66, 108.05)	73.41 (48.59, 112.35)	0.171
INS.1h, mU/L	76.22 (50.43, 120.1)	79.54 (50.97, 119.7)	74.91 (50.38, 120.1)	0.559
INS.2h, mU/L	60.26 (37.01, 99.82)	56.92 (35.24, 95.81)	62.03 (37.39, 102.02)	0.039
INS.3h, mU/L	25.26 (12.47, 46.9)	26.38 (11.26, 46.7)	24.7 (12.91, 46.94)	0.493
TCH, mmol/L	4.9 (4.21, 5.56)	4.9 (4.2, 5.58)	4.9 (4.21, 5.56)	0.707
TG, mmol/L	1.04 (0.73, 1.53)	1.01 (0.71, 1.46)	1.07 (0.74, 1.58)	0.081
Glu, mmol/L	4.85 (4.53, 5.19)	4.8 (4.54, 5.15)	4.87 (4.53, 5.2)	0.221
ApoE, mg/dl	4.59 (3.93, 5.42)	4.57 (3.94, 5.44)	4.61 (3.93, 5.41)	0.983
LPa, mg/L	122.5 (65, 264)	122.5 (60, 269.2)	122.5 (66, 263.78)	0.459

M, median; Q1, first quartile; Q3, third quartile.

### Feature selection

**Figure 2:**
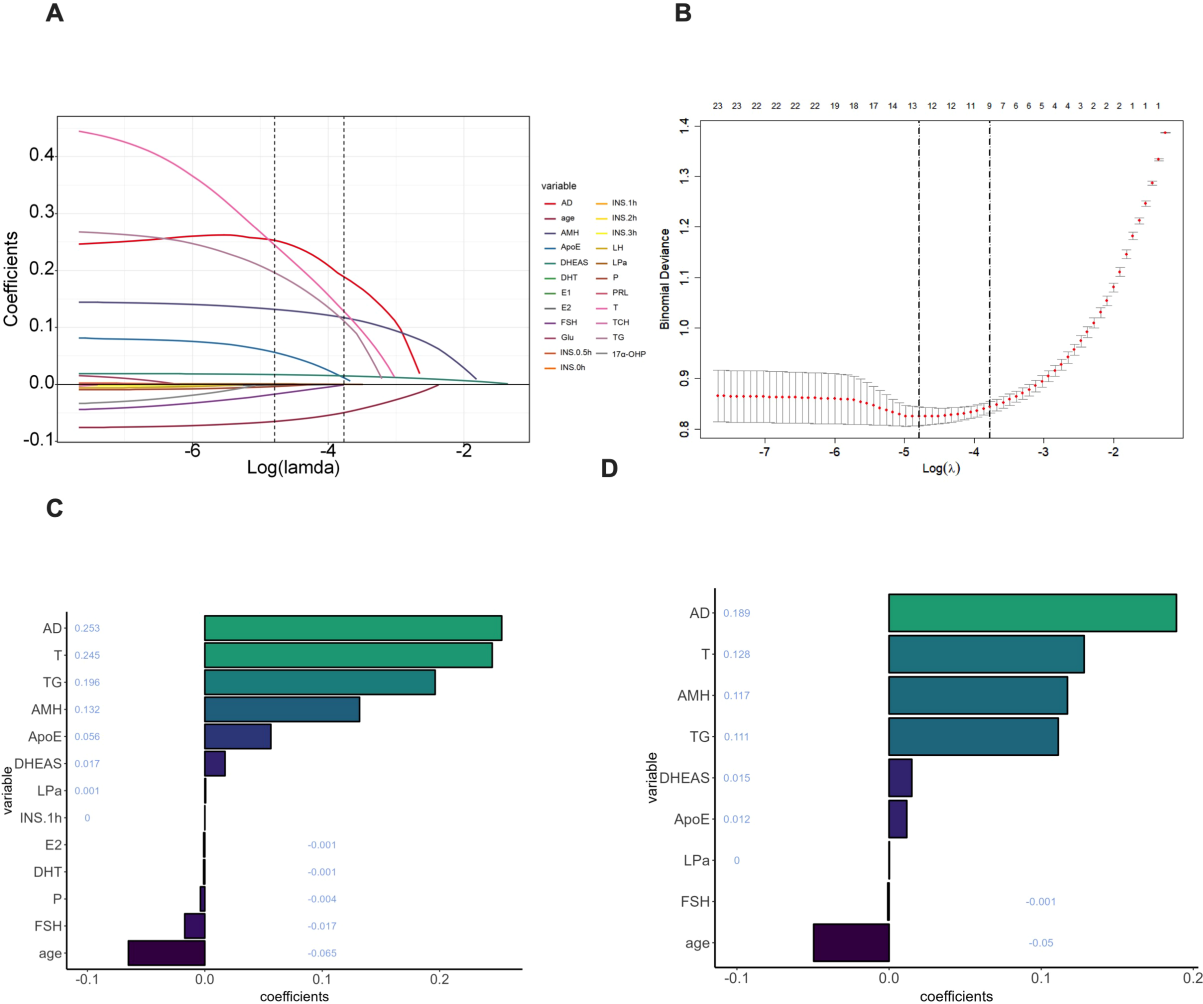
LASSO regression feature selection. **(A)** Coefficient profile plot; **(B)** Ten-fold cross-validation curve with designated λ.min and λ.1se; **(C, D)** Variables having nonzero coefficients. For model development, the following variables were chosen: AD, T, AMH, TG, DHEAS, ApoE, Lp(a), FSH, and age.

LASSO regression was used for feature selection after minimizing absolute shrinkage and selection. As shown in **Figure 2A**, the coefficient profiles indicate that most variable coefficients progressively shrank toward zero as the penalty parameter (λ) increased. **Figure 2B** displays the tenfold cross-validation results, where the two dashed lines represent the minimum error criterion (λ.min) and the one–standard error criterion (λ.1se). Under the λ.min criterion, nine variables with nonzero coefficients—AD, T, AMH, TG, DHEAS, ApoE, Lp(a), FSH, and age—were finally selected as key features strongly associated with the diagnosis of polycystic ovary syndrome (PCOS) (**Figure 2D**).

### Rad-score comparison

**Figure 3:**
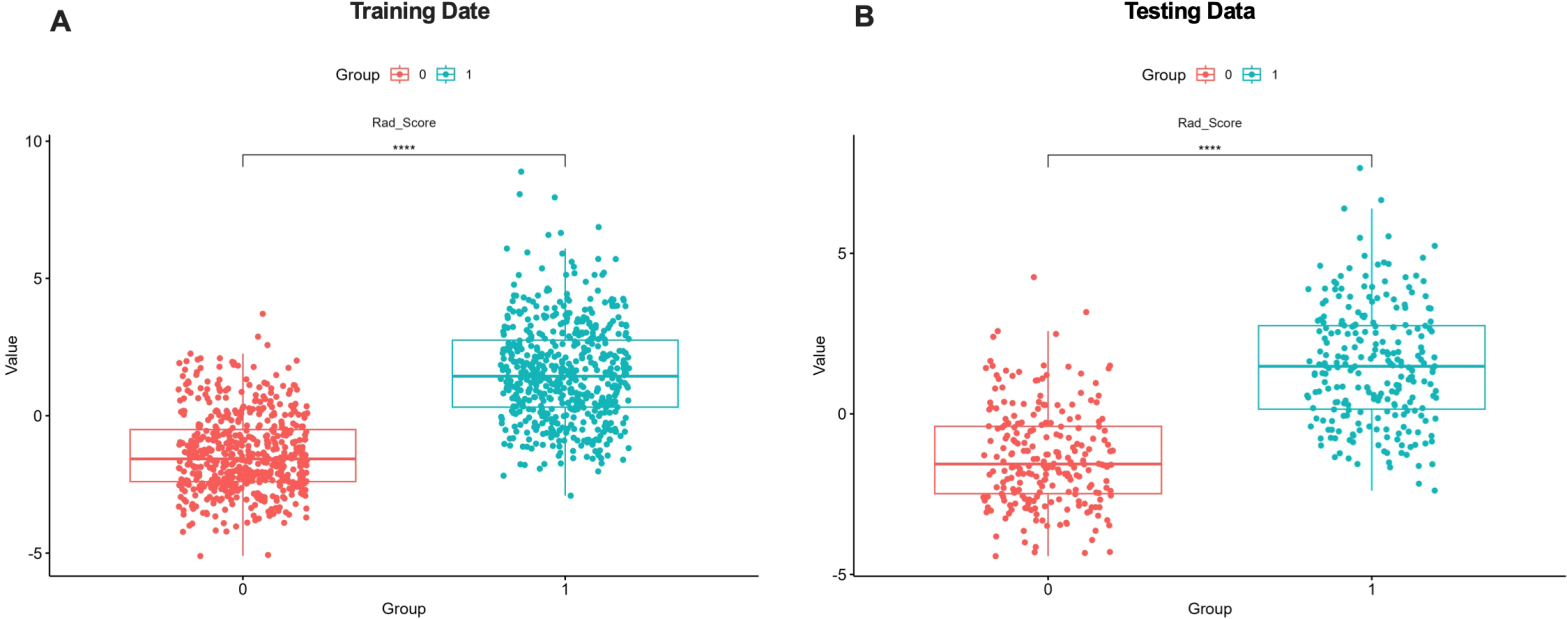
Comparison of Rad-scores between PCOS and non-PCOS groups. Rad-scores were significantly higher in the PCOS group (P< 0.0001). **(A)** Training Data; **(B)** Testing Data.

The Rad-score was calculated for each participant and compared between groups. As shown in **Figure 3**, patients with PCOS (label = 1) had significantly higher Rad-scores than those without PCOS (label = 0) (P< 0.0001), indicating good discriminative ability. The Rad-score may serve as a reliable marker for distinguishing PCOS from non-PCOS and holds potential value for early diagnosis and risk stratification.

### Waterfall plot

**Figure 4:**
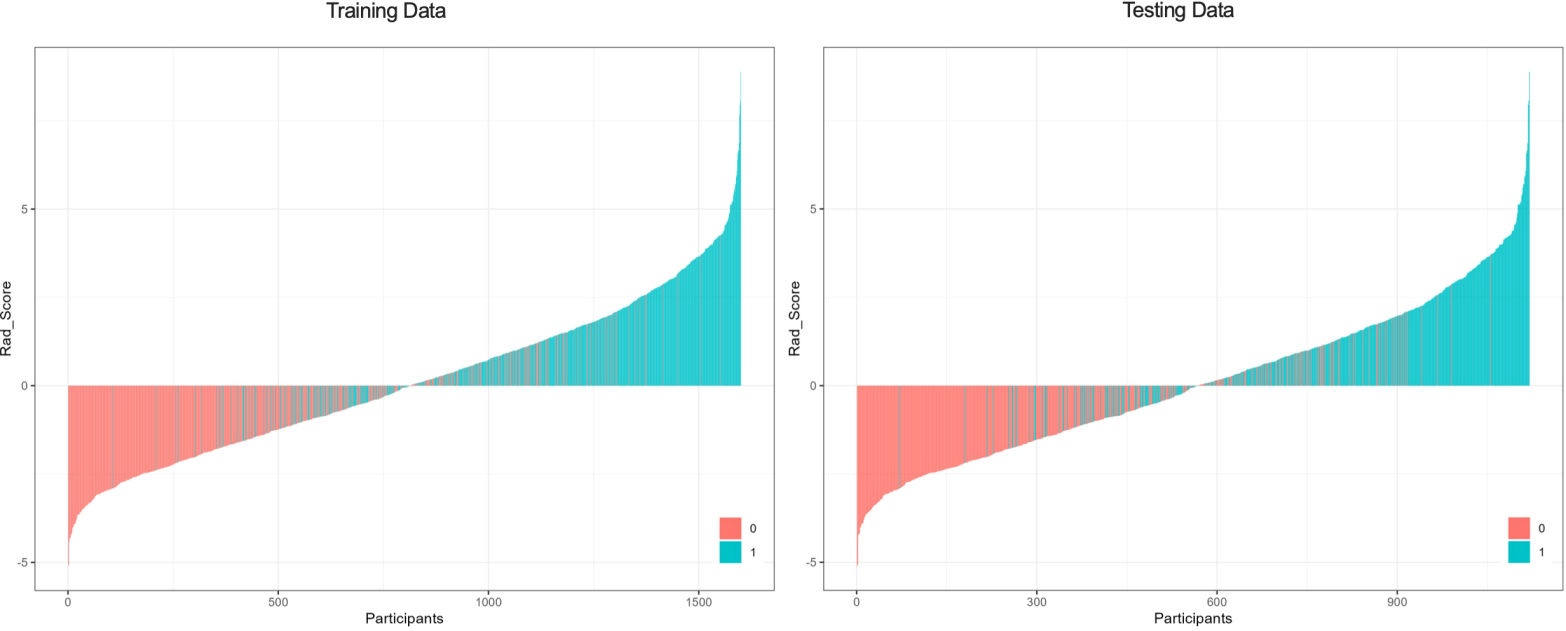
Waterfall plots of Rad-scores in the training and validation cohorts. Each bar represents an individual participant; blue indicates those with PCOS, and red indicates those without PCOS. Overall, PCOS patients showed higher Rad-scores, demonstrating the model’s strong discriminative ability. **(A)** Training Data; **(B)** Testing Data.

To further evaluate the model’s discrimination at the individual level, waterfall plots of Rad-scores were generated for the training and validation cohorts (**Figure 4**). Distinct distributions of Rad-scores were observed between patients with PCOS and those without PCOS in both cohorts, indicating that the model can consistently distinguish between the two groups.

### Model performance comparison

**Figure 5:**
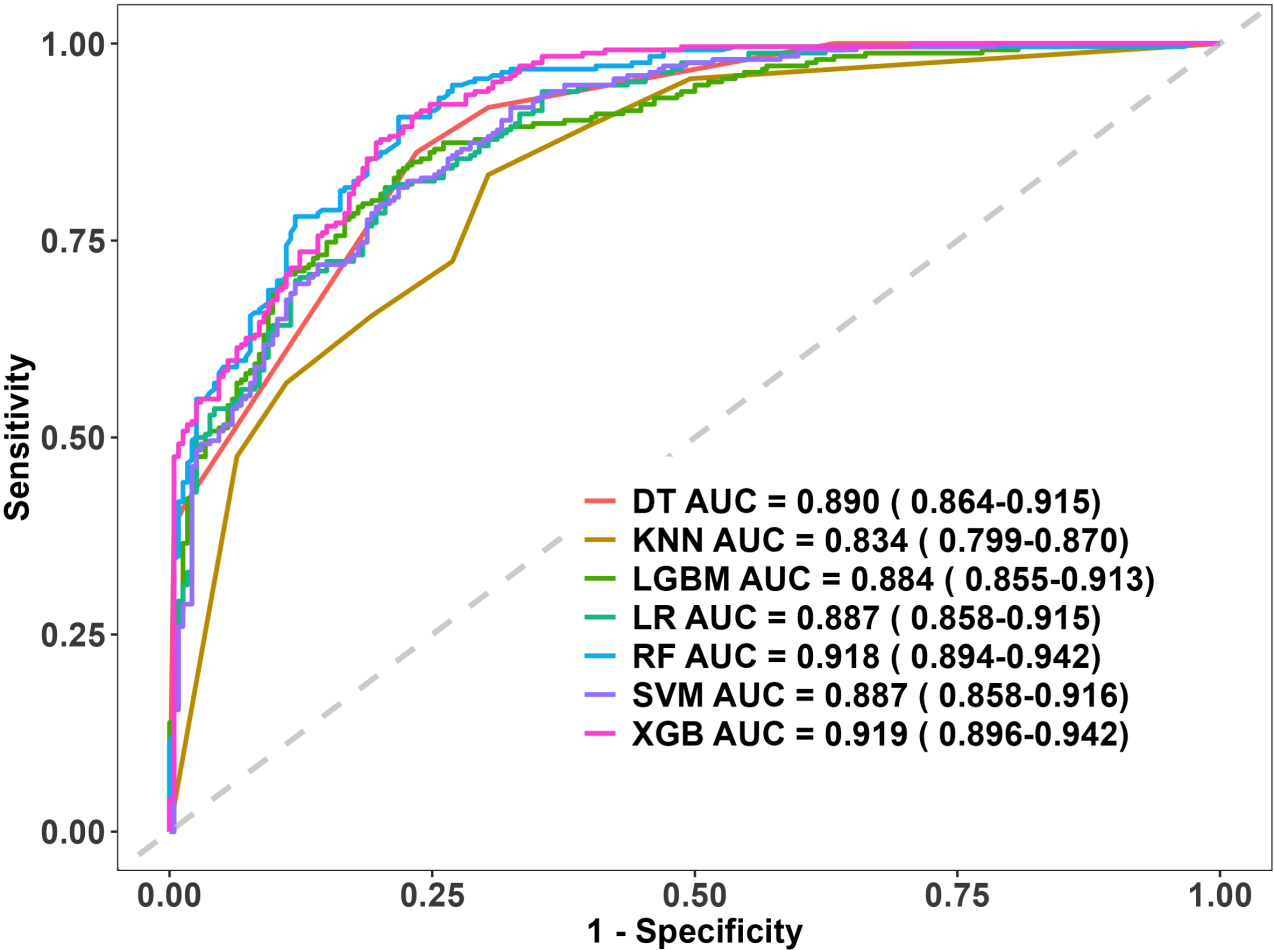
ROC curves for the machine learning models. LGBM, Light Gradient Boosting Machine; XGB (XGBoost), extreme gradient boosting; RF, random forest; DT, decision tree; SVM, support vector machine; KNN, k-nearest neighbors; LR, logistic regression; ROC, receiver operating characteristic; AUC, area under the curve.

The predictive performance of seven machine learning models was compared (**Figure 5**, **Table 2**). Among them, the XGBoost model achieved the best overall performance (AUC = 0.919, 95% CI: 0.896–0.942), demonstrating a better balance across metrics than the other algorithms. The Random Forest model ranked second (AUC = 0.918), while the K-Nearest Neighbors (KNN) model performed the poorest. Detailed performance metrics for all models are summarized in **Table 2**.

**Table 2:** PCOS prediction machine learning model performance.

Model	AUC	ACC	SEN	SPE	PPV	NP V	Recall	F1	Precision
DT	0.890	0.8146	0.7940	0.8404	0.8618	0.7650	0.7940	0.8265	0.8618
RF	0.918	0.8292	0.8455	0.8120	0.8254	0.8333	0.8455	0.8353	0.8254
XGB	0.919	0.8062	0.7805	0.8333	0.8312	0.7831	0.7805	0.8050	0.8312
SVM	0.887	0.7917	0.8740	0.7051	0.7570	0.8418	0.8740	0.8113	0.7570
KNN	0.834	0.7271	0.7236	0.7308	0.7386	0.7155	0.7236	0.7310	0.7386
LGBM	0.884	0.8021	0.8049	0.7991	0.8082	0.7957	0.8049	0.8065	0.8082
LR	0.887	0.8125	0.7237	0.8220	0.8116	0.7520	0.7350	0.7620	0.7650

PCOS, Polycystic Ovary Syndrome; LGBM, Light Gradient Boosting Machine; XGB, Extreme Gradient Boosting; RF, Random Forest; DT, Decision Tree; SVM, Support Vector Machine; KNN, K-Nearest Neighbors; LR, Logistic Regression; PPV, positive predictive value; NPV, negative predictive value; F1, F1 score.

### Calibration and clinical utility analysis

**Figure 6:**
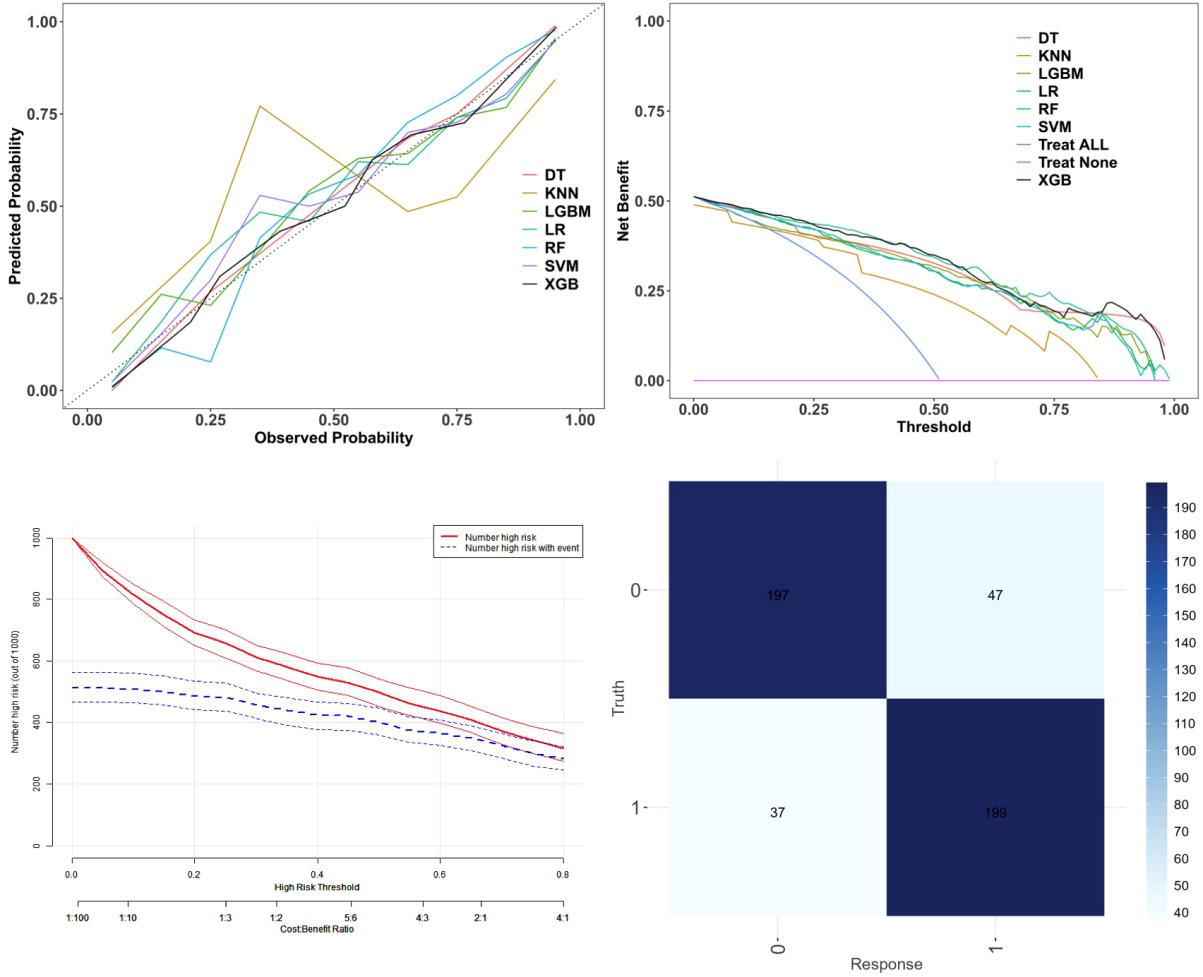
Calibration performance and clinical utility of the models. **(A)** calibration curves; **(B)** decision curve analysis (DCA); **(C)** clinical impact curve (CIC); **(D)** confusion matrix of the best-performing XGB model. DT, Decision Tree; XGB, Extreme Gradient Boosting; LGBM, Light Gradient Boosting Machine; RF, Random Forest; SVM, Support Vector Machine; KNN, K-Nearest Neighbors; LR, Logistic Regression.

Following the comparison of overall predictive performance, we further evaluated the calibration and clinical utility of the seven models (**Figure 6**).

(A) The calibration curves show how well the predicted probabilities align with the observed outcomes. Overall, the XGB model (black line) showed the closest fit to the ideal diagonal, indicating superior calibration. The RF, SVM, LGBM, DT, and LR models also demonstrated acceptable consistency, whereas the KNN model exhibited larger deviations across risk intervals, suggesting less reliable predictions. These findings confirm that XGB achieved the best balance between accuracy and consistency.

(B) The decision curve analysis (DCA) compared the net clinical benefit of the models across various threshold probabilities. All models outperformed the “Treat None” strategy (purple line), and most performed better than the “Treat All” strategy (blue curve) in the low-to-intermediate risk range, suggesting positive clinical value. Among them, the XGB model consistently maintained the highest net benefit across almost all thresholds, indicating superior capability in balancing false positives and false negatives. In contrast, KNN and SVM showed greater variability, reflecting lower stability. Collectively, these results support the XGB model as the most clinically applicable algorithm.

(C) The clinical impact curve (CIC) further assessed the XGB model’s ability to identify high-risk individuals at different thresholds. The red solid line represents the number of individuals predicted as high-risk, and the blue dashed line represents the actual number of true-positive cases, with 95% confidence intervals shown as shaded boundaries. As the threshold increased, the number of predicted high-risk cases declined, reducing false positives but potentially missing actual cases. Notably, at a threshold of 0.5, the two curves nearly overlapped, and the confidence interval narrowed, indicating optimal agreement between predicted and observed outcomes. This threshold provided the optimal trade-off between sensitivity and specificity, thereby maximizing the benefits of clinical intervention. The CIC thus quantitatively demonstrates the clinical applicability of the XGB model for individualized risk assessment of PCOS.

The confusion matrix of the XGB model is presented in **Figure 6D**, offering a visual evaluation of its classification performance.

### External validation

**Figure 7:**
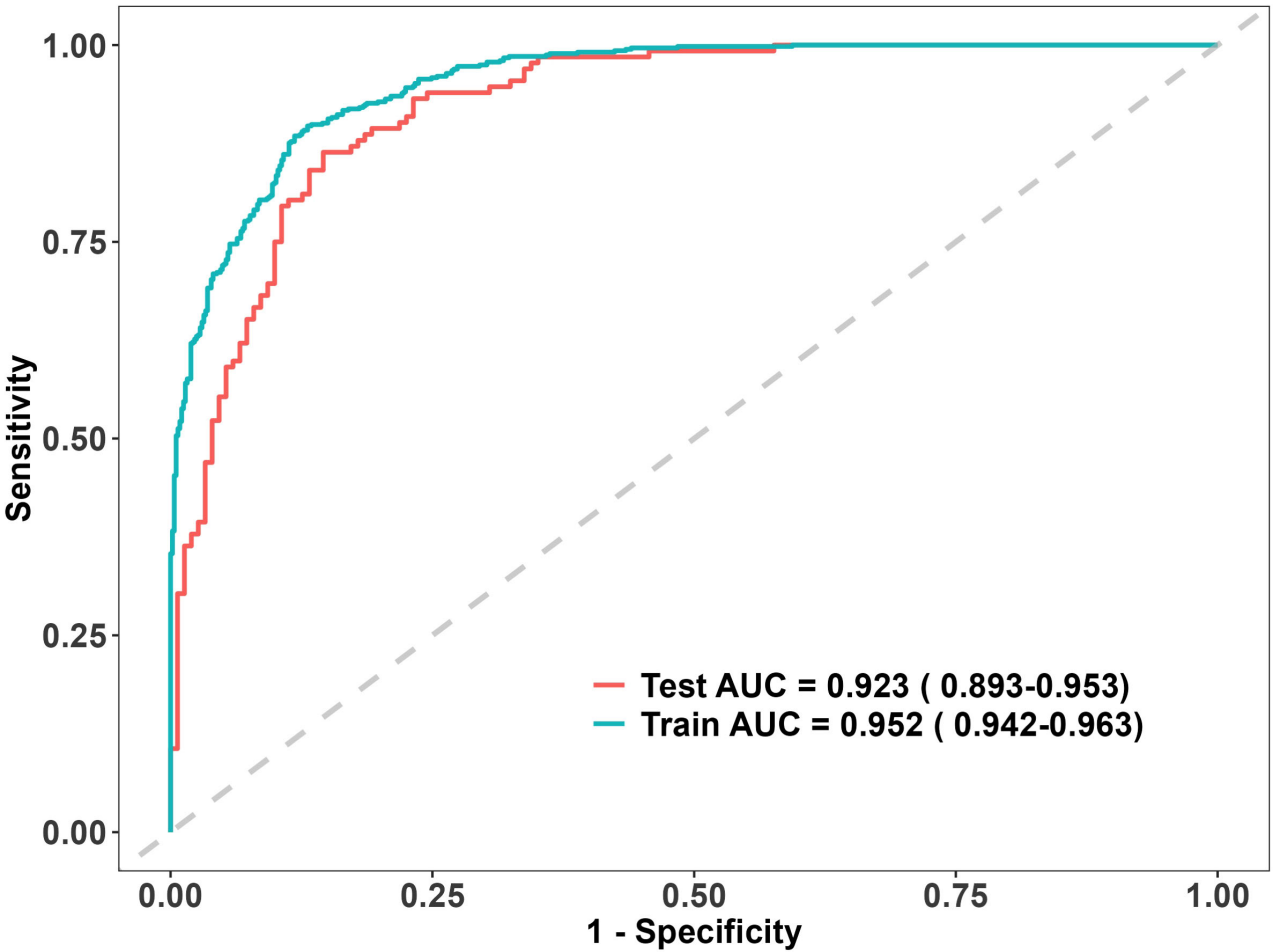
External validation in an independent cohort.

The proposed XGBoost model demonstrated strong generalizability. In **Figure 7**, the ROC curve for the external validation cohort showed an AUC of 0.923 (95% CI: 0.893–0.953), indicating strong predictive ability.

### Model interpretability analysis

**Figure 8:**
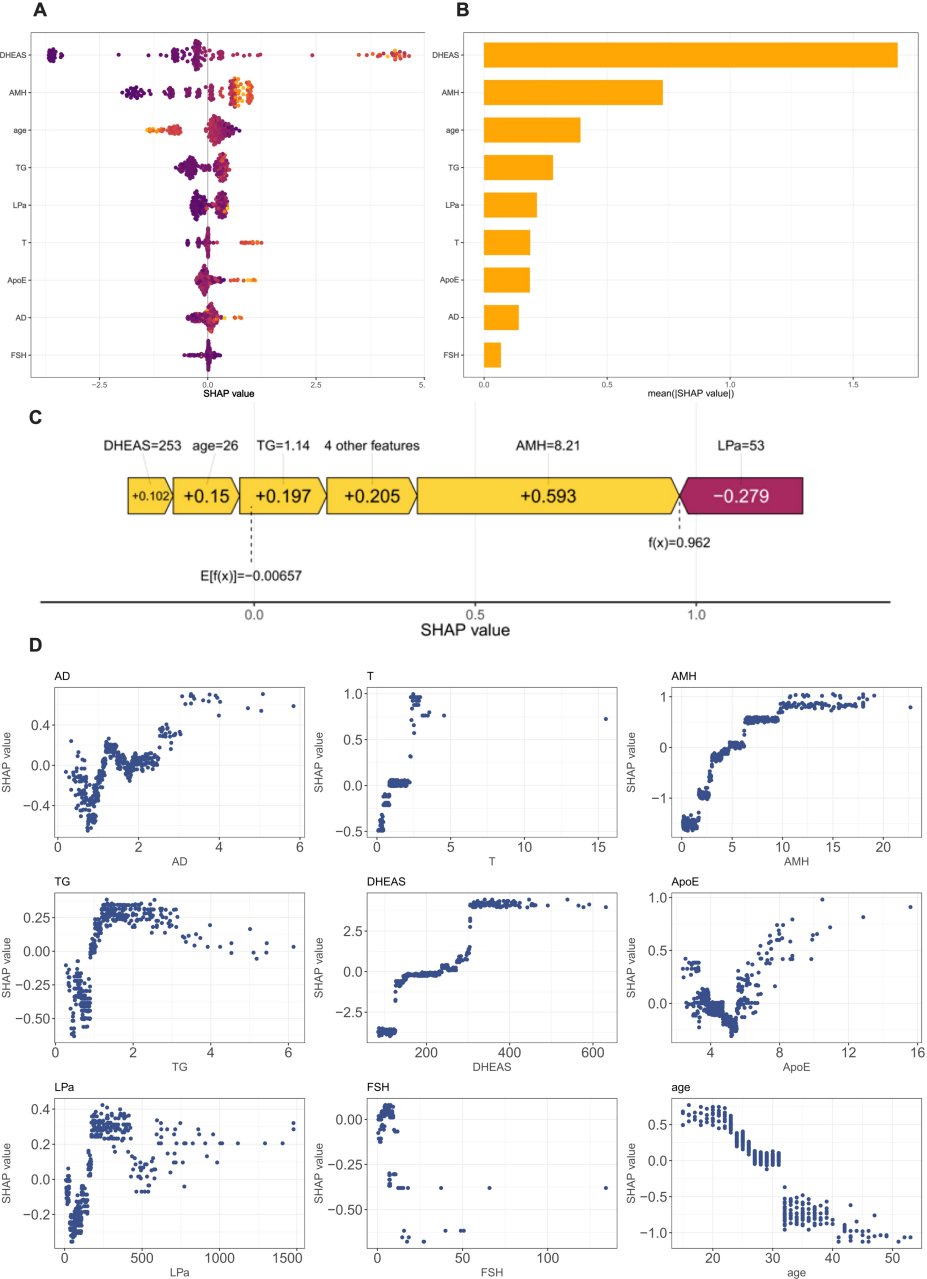
Global and local model interpretation using SHAP analysis. **(A)** SHAP beeswarm summary plot showing the distribution and direction of feature contributions across all samples. Each dot represents one patient, colored by the feature value (yellow = high, purple = low). Features at the top have a greater overall impact on the model output. **(B)** Bar plot of mean absolute SHAP values indicating the overall importance of each variable. Together, **(A, B)** show that DHEAS and AMH were the most influential positive predictors, followed by TG and age, whereas Lp(a) had a substantial negative contribution. The effects of T, ApoE, AD, and FSH were relatively minor. **(C)** SHAP waterfall plot for an individual case illustrating that AMH, TG, and age were major positive contributors, while Lp(a) was the primary negative contributor. **(D)** SHAP main effect dependence plots of dominant features predicting PCOS. DHEAS and AMH exerted the most potent positive effects on PCOS prediction, while metabolic markers (ApoE and Lp(a)) showed a moderate influence. FSHand age were negatively associated with PCOS, consistent with its hormonal and epidemiological characteristics. Each point represents a single patient, where the x-axis indicates the actual feature value and the y-axis represents its SHAP value. Positive SHAP values push the prediction toward PCOS.

With the growing application of artificial intelligence in medicine, model interpretability has become an important indicator for evaluating the usability and safety of predictive models (29). Doshi-Velez and Kim et al. emphasized that interpretability should be regarded as a core component of machine learning science and systematically assessed in high-risk domains (30).To measure the contribution of each feature to model predictions, we employed the Shapley Additive exPlanations (SHAP) approach (**Figure 8**). SHAP, a game theory–based feature attribution method, is one of the most widely used interpretability algorithms (31).

At the global level, the beeswarm and bar plots (**Figures 8A, B**) revealed the relative importance of nine key predictors. DHEAS and AMH were the most influential positive contributors, with higher values significantly increasing SHAP scores and the predicted probability of PCOS. TG and age also showed positive associations, but with lower and more heterogeneous contributions across individuals. In contrast, elevated Lp(a) values were predominantly distributed in the negative SHAP region, indicating an inhibitory effect on prediction. FSH showed an overall negative association, consistent with the typical hormonal profile of PCOS, characterized by an elevated LH/FSH ratio. These findings suggest that PCOS development involves not only reproductive hormonal dysregulation but also metabolic disturbances, reflecting its potential pathophysiological heterogeneity.

At the individual level, the waterfall plot (**Figure 8C**) illustrated the cumulative contribution of features to a single patient’s prediction. The baseline prediction value of 0.00657 increased to 0.962 after integrating all feature effects, indicating that the case was classified as high risk. AMH (+0.593), TG (+0.197), age (+0.15), and DHEAS (+0.102) were the main positive drivers, while Lp(a) (–0.279) acted as the primary negative factor. This suggests that multiple hormonal and metabolic factors jointly contributed to the patient’s high-risk prediction.

The dependence plots (**Figure 8D**) revealed several threshold effects. Specifically, AMH values above five ng/mL shifted from neutral to strongly positive contributions, and levels exceeding 10 ng/mL almost deterministically indicated PCOS. DHEAS exhibited a substantial positive contribution around 200 μg/dL, which further increased with concentration, suggesting a dose–response relationship. FSH showed positive SHAP values at lower levels but negative contributions at higher levels, aligning with the characteristic lower FSH levels in patients with PCOS. Moreover, a younger age (<30 years) was positively associated with PCOS prediction, consistent with its known epidemiological pattern.

### Nomogram construction and validation

**Figure 9:**
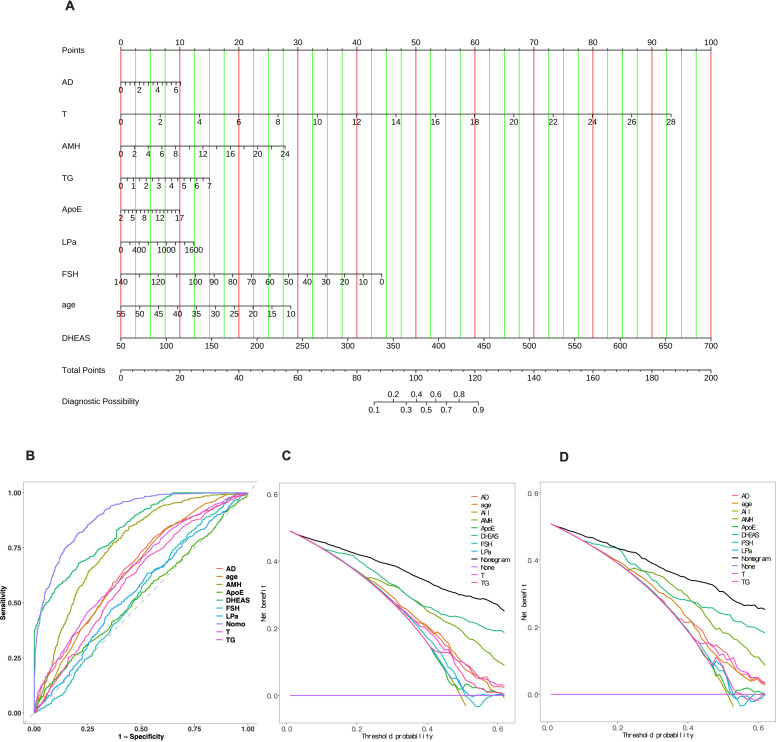
Nomogram and evaluation of its diagnostic performance and clinical utility. **(A)** Nomogram for predicting PCOS diagnosis. **(B)** Comparison of ROC curves between individual predictors and the nomogram model. **(C)** Decision curve analysis (DCA) for the train cohort. **(D)** Decision curve analysis (DCA) for the test cohort.

SHAP analysis not only enhanced model transparency but also helped identify the most clinically relevant predictors. However, SHAP plots are primarily suited for research interpretation, whereas clinicians require intuitive and straightforward tools for individualized risk assessment. Therefore, based on the key variables identified by the XGBoost model, we developed a simplified logistic regression–based nomogram (**Figure 9A**). Using a few routine indicators, clinicians can easily calculate the total score and estimate an individual’s likelihood of having PCOS.

In clinical practice, the nomogram provides physicians with an intuitive and easy-to-use tool to quickly assess a patient’s risk of having PCOS based on routine clinical data. For example, suppose a patient’s clinical data is as follows: age 30 years, AMH 10 ng/mL, FSH 60, triglycerides (TG) 5, ApoE 12, and DHEAS 150. By inputting these data into the nomogram, we calculate the corresponding points for each variable: AD 2, points = 3; T 6, points = 20; AMH 10, points = 12; TG 5, points = 10.5; ApoE 12, points = 6.5; FSH 60, points = 25; Age 30 years, points = 16.75; DHEAS 150, points = 15; Lp(a) 1000, points = 7.5. By summing these points, we obtain a total points of 116.25. Based on this total score of 116.25, the corresponding PCOS risk for this patient is 82.5%. This result indicates that the patient has a high risk of PCOS, and the clinician can use this information to decide whether to perform further diagnostic tests, such as ultrasound or hormonal assessments, or adjust the treatment plan. With the nomogram, physicians can quickly and intuitively assess the patient’s risk, assist in developing personalized treatment plans, and enhance clinical decision-making efficiency, especially in resource-limited settings.

The ROC curves showed that the nomogram achieved better discrimination than individual predictors (**Figure 9B**), demonstrating strong predictive performance (AUC = 0.901 for the train set and 0.887 for the test set; **Table 3**). The decision curve analysis (DCA) further indicated higher net clinical benefit across most threshold probabilities in both cohorts (**Figures 9C, D**). Other performance metrics, including sensitivity, specificity, PLR, NLR, and Kappa values, remained stable between datasets (**Table 3**), supporting the model’s robustness and clinical utility.

**Table 3:** Performance metrics of the nomogram in the train and test sets.

Data set	AUC(95%CI)	ACC	SEN	SPE	NLR	PLR	PPA	NPA	TPA	KAPPA
Trian set	0.901(0.884,0.918)	0.815	0.778	0.852	0.261	5.242	0.778	0.852	0.815	0.63
Trian set binary	0.814(0.782,0.846)	0.814	0.778	0.85	0.261	5.18	0.778	0.85	0.814	0.628
Test set	0.887(0.858,0.915)	0.804	0.813	0.795	0.235	3.963	0.813	0.795	0.804	0.608
Test set binary	0.776(0.724,0.828)	0.775	0.728	0.825	0.33	4.153	0.728	0.825	0.775	0.551

**Figure 10:**
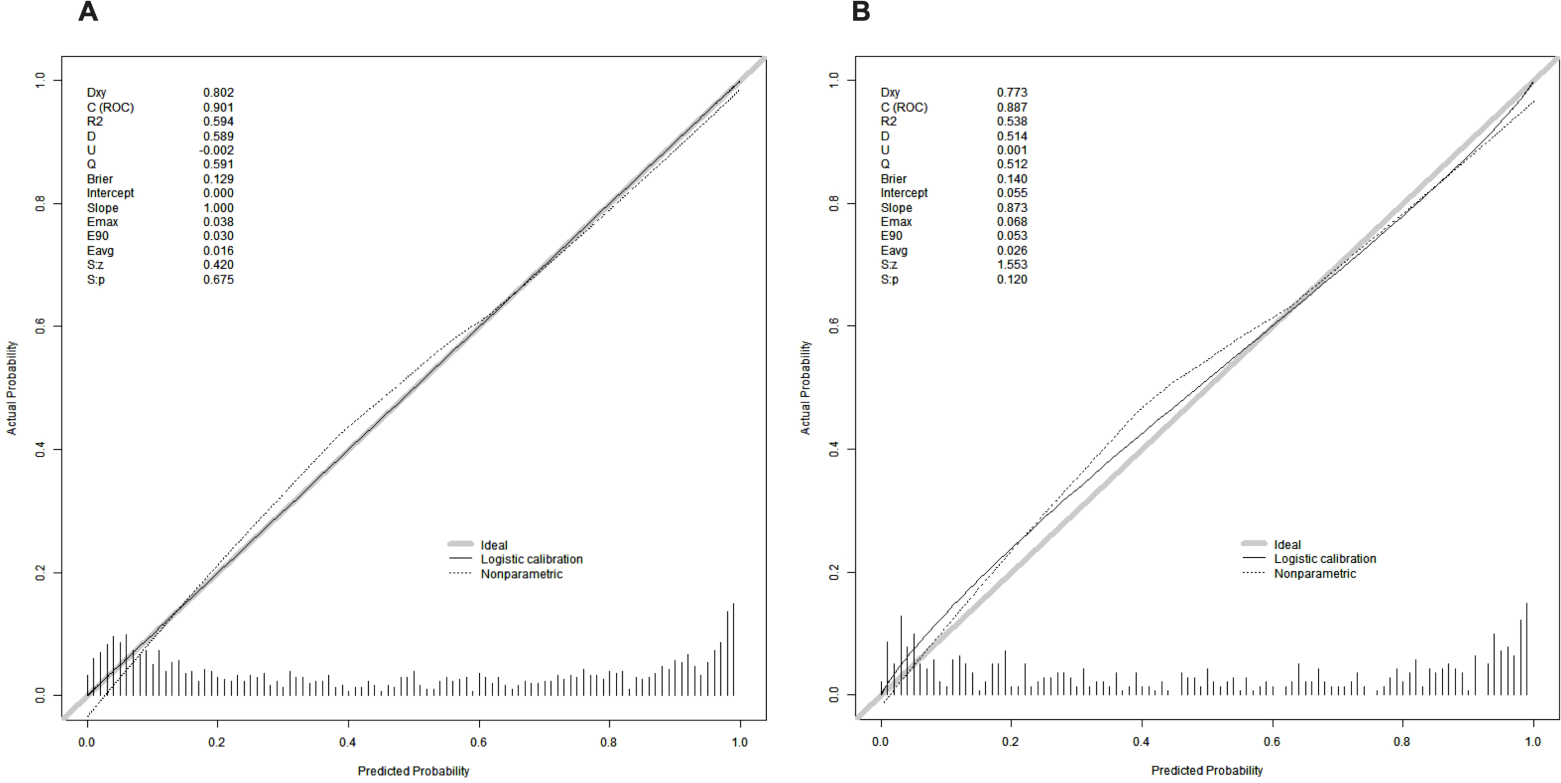
Calibration curves of the nomogram: **(A)** train cohort; **(B)** test cohort. The diagonal line represents perfect prediction, the solid line represents logistic calibration, and the dotted line shows the nonparametric calibration.

To further assess the model’s goodness of fit, calibration curves were plotted (**Figure 10**). The predicted probabilities showed good agreement with the observed outcomes in both the train and test cohorts, with the curves closely following the ideal diagonal line. The Brier score, Emax, and Eavg were 0.129, 0.038, and 0.016 in the train cohort, and 0.140, 0.068, and 0.026 in the test cohort, respectively, demonstrating excellent calibration performance.

Together, the XGBoost-based framework and its simplified nomogram demonstrated consistent and reliable performance across datasets, laying the groundwork for further clinical validation and broader application.

## Conclusion

An interpretable XGBoost model and a simplified nomogram were developed using routine hormonal and metabolic indicators. This dual-model approach shows strong potential for early detection of PCOS, pending further validation in larger prospective studies.

## Discussion

In this study, we developed an interpretable XGBoost model based on routine hormonal and metabolic indicators to predict PCOS. The model demonstrated excellent and stable performance across datasets, achieving an AUC of 0.919in the training cohort and 0.923 in the external validation cohort, outperforming all other machine learning algorithms. The Rad-score effectively discriminated between PCOS and non-PCOS cases and maintained stability across cohorts, while waterfall plots confirmed a strong individual-level discriminative ability. Furthermore, the decision curve analysis (DCA) and clinical impact curve (CIC) consistently indicated a higher net clinical benefit over a wide range of thresholds, and calibration curves demonstrated strong agreement between predicted and observed probabilities. Collectively, these findings confirm that the proposed model possesses robust predictive accuracy, good calibration, and high clinical reliability.

Using SHAP analysis, we quantified feature contributions and identified biologically meaningful threshold effects. AMH, DHEAS, and testosterone emerged as the most influential positive predictors, consistent with the hormonal profile characteristic of PCOS. Triglycerides (TG) and ApoE also contributed positively, suggesting the presence of concurrent metabolic abnormalities. Conversely, FSH, Lp(a), and age exerted adverse effects, aligning with the clinical pattern that PCOS predominantly affects younger women with lower FSH levels. The inverse association between age and PCOS risk is consistent with previous epidemiological findings showing a decline in androgen levels and an increase in metabolic disturbances with advancing age (32). These results emphasize that PCOS development involves a complex interplay between reproductive hormones and metabolic dysregulation.

Model interpretability is fundamental for fostering clinicians’ trust and ensuring the safe integration of AI-based tools in medical practice. Although SHAP provides valuable insight into feature-level contributions, its visualization and analytical depth are better suited to research contexts than to routine clinical workflows. Prior studies have highlighted that clinicians prioritize clarity, usability, and clinical relevance when interpreting AI outputs; however, current explainable AI (XAI) frameworks remain limited in bridging this gap (33). Future work should focus on refining interpretable visualization approaches and developing user-oriented explanations that better integrate with clinical reasoning, thereby facilitating the broader adoption of AI-assisted decision support systems (34).

In comparison to existing literature, our study offers several notable strengths. Previous work has predominantly focused on the diagnostic value of AMH alone. Van der Ham et al. in Fertility and Sterility and Gomes et al. in AJOG both confirmed AMH as a reliable biomarker for PCOS diagnosis (35, 36). The latest international guideline (Teede et al., 2023, JCEM) further incorporated AMH as an alternative to PCOM in adult diagnostic criteria, while cautioning against its use as a standalone test, especially in adolescents (10). However, most of these studies relied on single-variable or traditional regression models. Attempts to integrate multiple biomarkers—such as the model proposed by Tong et al. (37)—were limited by small sample sizes and a lack of external validation. In imaging studies, Moral et al. achieved high diagnostic accuracy using deep learning on ultrasound images; however, their model’s reliance on imaging quality limits its clinical generalizability (38). By contrast, our multicenter, large-sample study employed multi-model comparison and independent validation, addressing these shortcomings. Moreover, SHAP analysis identified clinically interpretable thresholds, such as an AMH level greater than five ng/mL, which is consistent with current international guidelines and evidence.

The proposed dual-model strategy, combining an interpretable XGBoost model with a simplified logistic regression–based nomogram, provides a practical balance between predictive accuracy and clinical usability. This approach is particularly advantageous for identifying early PCOS risk in resource-limited settings, enabling frontline clinicians to conduct personalized risk assessments and interventions. However, it is important to acknowledge that anthropometric parameters, such as body mass index (BMI) and waist circumference, which are key determinants of PCOS, were not included in this study. These variables, commonly used in clinical practice, could further enhance the model’s predictive accuracy, particularly in assessing metabolic risks and obesity-related complications. Future research should consider incorporating these anthropometric measurements to improve the comprehensiveness and precision of PCOS screening tools.

The nomogram, as an intuitive risk assessment tool, can quickly calculate disease risk based on routine clinical data from patients. However, to effectively integrate the nomogram into clinical practice, it must first be connected to the electronic health record (EHR) system, which will automatically retrieve patient data and calculate risk scores. This tool can serve as a decision support system, helping doctors quickly assess patient risk and develop personalized treatment plans. To ensure effective application, it is necessary to train doctors and regularly evaluate and optimize the model to improve its clinical adaptability and accuracy. In this study, we demonstrated how to use the nomogram to assess the risk of PCOS by inputting clinical data such as age, AMH, FSH, and calculating the score for each variable to determine the individual PCOS risk. This method provides clinicians with a simple and effective tool that helps them make faster and more accurate decisions.

Nevertheless, several limitations should be acknowledged. First, as a retrospective study, potential selection bias cannot be excluded. Although validated externally, as Van Calster et al. (39)noted, validation is not the endpoint, and temporal or population-based generalizability warrants further investigation. Second, despite the inclusion of multicenter data, the overall sample size remains moderate; future large-scale, prospective studies are needed to confirm model stability. Third, this study did not incorporate imaging or multi-omics data (e.g., metabolomics, microbiomics). Recent studies have reported significant alterations in the gut microbiota and its metabolites among women with PCOS, suggesting that the gut–metabolic axis may play a key role in disease pathogenesis (40). Future research integrating multi-dimensional data in prospective designs may further enhance the robustness, interpretability, and translational potential of this dual-model framework.

Since all participants in this study were from China, the generalizability of the findings to other ethnic or geographical populations may be limited. Ethnic and geographical factors, such as genetic variations, environmental influences, and healthcare access, may contribute to differences in disease presentation and risk factors. Future studies should consider incorporating multi-ethnic and multi-regional cohorts to validate the applicability and generalizability of the model across diverse population.

Additionally, it is important to consider the impact of menstrual cycle phase and assay timing on the accuracy of hormonal measurements. Hormones such as FSH, AMH, and LH fluctuate at different stages of the menstrual cycle, especially during the follicular and luteal phases, where FSH and LH levels can vary significantly. To minimize these fluctuations, we recommend that hormonal measurements be performed during standardized phases of the menstrual cycle, such as days 2 to 5 of menstruation. Future studies should further standardize assay timing to ensure that data are collected during consistent menstrual cycle phases, thus improving the stability and accuracy of predictive models.

## Data Availability

The original contributions presented in the study are included in the article/supplementary material. Further inquiries can be directed to the corresponding authors.
